# No increase in translocated chromosomal aberrations, an indicator of ionizing radiation exposure, in childhood thyroid cancer in Fukushima Prefecture

**DOI:** 10.1038/s41598-023-41501-x

**Published:** 2023-08-31

**Authors:** Akira Sakai, Naohiro Tsuyama, Tetsuya Ohira, Misaki Sugai-Takahashi, Takashi Ohba, Yusuke Azami, Yoshiko Matsumoto, Iwadate Manabu, Satoshi Suzuki, Maki Sato, Mitsuaki Hosoya, Tetsuo Ishikawa, Shinichi Suzuki

**Affiliations:** 1 Department of Radiation Life Sciences, Fukushima Medical University School of Medicine, 1 Hikarigaoka, Fukushima, 960-1295 Japan; 2 Department of Epidemiology, Fukushima Medical University School of Medicine, Fukushima, Japan; 3 Department of Radiological Sciences, Fukushima Medical University School of Health Sciences, Fukushima, Japan; 4 Department of Radiation Oncology, Southern Tohoku General Hospital, Sendai, Japan; 5 Department of Thyroid and Endocrinology, Fukushima Medical University School of Medicine, Fukushima, Japan; 6 Department of Pediatrics, Fukushima Medical University School of Medicine, Fukushima, Japan; 7 Department of Radiation Physics and Chemistry, Fukushima Medical University School of Medicine, Fukushima, Japan; 8 Department of Thyroid Treatment, Fukushima Medical University School of Medicine, Fukushima, Japan

**Keywords:** Epidemiology, Cytogenetics

## Abstract

To investigate the effects of radiation exposure due to the Fukushima nuclear power plant accident, following the disaster Fukushima Prefecture launched thyroid ultrasound examinations of residents who were generally younger than 18 years at the time of the earthquake. As the rate of pediatric thyroid cancer was higher than expected, we conducted biological dose assessment based on the frequency of translocated chromosome (Tr) aberrations using peripheral blood lymphocytes. Tr formation frequency was compared among the thyroid cancer (n = 38, median age 18 years, age range 12–26 years), thyroid-related disease (n = 30, median age 21 years, age range 15–28 years), and healthy controls (n = 31, median age 22 years, age range 20–23 years) groups. Tr aberration frequency was initially significantly higher in the thyroid cancer than in the other two groups; however, differences among the groups disappeared after adjusting for history of CT scan, as 92%, 67%, and 28% of those in the thyroid cancer, thyroid-related disease, and control groups, respectively, had undergone CT previously. Therefore, the significant difference in the initial number of Tr formations is presumably due to radiation exposure from CT. Accordingly, the effects of medical exposure on the chromosomes of children and adolescents should be noted.

## Introduction

The Great East Japan Earthquake (GEJE) of 11 March, 2011 and the subsequent tsunami caused an accident at the Fukushima Daiichi Nuclear Power Plant that resulted in widespread of radioactive contamination in Fukushima Prefecture (FP). After the accident at the Chernobyl Nuclear Power Plant in 1986, there was an increase in cases of pediatric thyroid cancer due to internal radiation exposure, which is characterized by a 4–5 year latent period followed by a rapid increase in incidence^1–4^. Therefore, primary examination of the Thyroid Ultrasound Examination (TUE) program conducted in FP in 2011–2013, and included 367,649 subjects (81.7% coverage)) who were generally ≤ 18 years old and living in FP at the time of the disaster. Conducting the TUE immediately after the GEJE enabled comparison of the obtained results with subsequent occurrence of thyroid cancer (5). Thereafter, primary examinations of TUE were conducted every two years until the examined individual was ≥ 20 years, with milestone examinations conducted every five years thereafter. As of 30 June, 2021, 263 of the examined individuals were found to have malignant or suspected malignant^5^.

The prevalence of thyroid cancer in Japan can be estimated from its incidence in people aged 15–19 years, which is 1.2 per 100,000 men and 3.3 per 100,000 women^6^. The number of patients in FP diagnosed with thyroid cancer through the TUE is clearly high. However, no significant differences in the incidence rate of thyroid cancer has been found between regions within FP, and no association has been identified between external radiation dose and the prevalence of thyroid cancer^5,7–11^. It has therefore been speculated that the increase in the incidence of thyroid cancer in FP after the GEJE is due to the effect of aggressive screening by the pediatric TUE^12,13^. As of 1 August, 2022, thyroid equivalent doses (the sum of internal and external doses) could be estimated based on the GEJE behavioral survey for 108 individuals (41.1% of patients with thyroid cancer), with a median value of 2.2 mSv (range 0.11–22.70)^14^.

However, biological dose assessment has not been performed for each thyroid cancer patient, and it has been 12 years since the nuclear accident, which makes it very difficult to estimate the dose to the thyroid gland. As alternative techniques, two biological dosimetry methods employ biomarkers of chromosome damage biomarker of peripheral bloods (PB) at the time of ionizing radiation exposure: one is based on the number of dicentric chromosomes (Dic), which is the international gold standard for acute exposure; and the other is based on the number of translocated chromosomes (Tr), which are stable type chromosomes, for chronic exposure^15^. In the case that several years have passed since radiation exposure, the latter method can be used to evaluate the effective dose, which is the dose to the whole body. For this purpose, dose–response curves are required for each institution. We have generated response curves in the low-dose range (8 doses: 0–1000 mGy) for Dic and Tr analysis from the PB of five healthy subjects^16^. We have also showed an increase in the number of Dic formations after a single CT scan examination^17,18^ and reported that it is difficult to find significant changes in the number of Tr formations after a single CT examination^18,19^.

In this study, we analyzed the number of Tr formations among patients with thyroid cancer, patients with thyroid-related disease (non-thyroid cancer), and healthy individuals of the same age as control in FP, with the aim of elucidating whether the occurrence of thyroid cancers detected in the TUE program is related to radiation exposure.

## Results

### Subject background data

Of 38 individuals diagnosed with thyroid cancer, 34 had papillary carcinoma and 4 had follicular carcinoma (Table 1). Thirty-five of the 38 (92%) patients had undergone a CT examination prior to PB collection (Fig. 2A), including 5 who had undergone CT more than once. In most of these patients, the scan sites were the neck and chest. In three patients, the scan range was from the neck to abdomen or from the neck to pelvis, and CT of the head was performed in one patient (Table 1).

**Table 1:** Patients with thyroid cancer.

Case no.	Pathology^#1^	Analyzed cells number	Cell equivalent (CE)	Observed translocation (Tr)	Two-way (reciprocal) translocation	One-way (non-reciprocal) translocation	Tr frequency [/100CE]	Age correction factor^#2^	Age-adjusted Tr frequency [/100CE]^#3^	Number of CT examination before analysis	CT examination sites^#4^
1	P	5297	2063.5	16	13	3	0.775	0.200	0.576	1	N, C
2	P	5315	2070.5	19	18	1	0.918	0.187	0.718	1	N, C
3	P	5262	2049.9	10	8	2	0.488	0.175	0.313	1	N, C
4	P	5278	2056.1	9	8	1	0.438	0.294	0.138	1	N, C
5	P	5228	2036.6	13	9	4	0.638	0.187	0.443	1	N, C
6	P	5303	2065.8	21	19	2	1.017	0.212	0.791	2	N, C/N, C
7	P	5207	2028.4	21	16	5	1.035	0.239	0.783	1	N, C
8	P	5321	2072.8	12	7	5	0.579	0.252	0.327	1	N, C
9	P	5362	2088.8	14	11	3	0.670	0.239	0.423	0	
10	P	5334	2077.9	15	9	6	0.722	0.280	0.432	1	N, C
11	P	5247	2044.0	21	5	16	1.027	0.225	0.802	1	N, C, A
12	P	5267	2051.8	20	12	8	0.975	0.225	0.737	1	N, C
13	P	5242	2042.1	22	13	9	1.077	0.294	0.769	1	N, C
14	F	5280	2056.9	19	9	10	0.924	0.225	0.698	1	N, C
15	P	5283	2058.0	18	13	5	0.875	0.266	0.597	3	N/N, C/C, A, P
16	P	5291	2061.2	23	15	8	1.116	0.309	0.807	1	N, C, A, P
17	F	5253	2046.4	4	2	2	0.195	0.324	0.000	0	
18	F	5263	2050.3	23	12	11	1.122	0.309	0.813	0	
19	F	5263	2050.3	19	16	3	0.927	0.309	0.618	1	N, C
20	P	5225	2035.4	24	16	8	1.179	0.212	0.951	1	N, C
21	P	5250	2045.2	18	12	6	0.880	0.239	0.642	1	N, C
22	P	5288	2060.0	15	12	3	0.728	0.175	0.553	3	N, C/N, C/N, C
23	P	5253	2046.4	17	13	4	0.831	0.294	0.536	2	N, C/N, C
24	P	5390	2099.7	20	13	7	0.953	0.266	0.687	1	N, C
25	P	5314	2070.1	15	10	5	0.725	0.163	0.562	1	H
26	P	5231	2037.8	12	7	5	0.589	0.225	0.364	1	N, C
27	P	5423	2112.6	28	21	7	1.325	0.163	1.145	1	N, C
28	P	5419	2111.0	23	16	7	1.090	0.280	0.810	1	N, C
29	P	6000	2337.4	9	5	4	0.385	0.294	0.091	1	N, C
30	P	5545	2160.1	12	9	3	0.556	0.355	0.201	1	N, C
31	P	5272	2053.8	10	9	1	0.487	0.239	0.248	1	N, C
32	P	5267	2051.8	28	24	4	1.365	0.339	1.026	1	N, C
33	P	5304	2066.2	30	19	11	1.452	0.225	1.227	1	N, C
34	P	5232	2038.2	14	8	6	0.687	0.212	0.465	1	N, C
35	P	5294	2062.3	39	28	11	1.891	0.212	1.654	1	N, C
36	P	5305	2066.6	19	15	4	0.919	0.187	0.720	1	N, C
37	P	5304	2066.2	18	10	8	0.871	0.225	0.646	1	N, C
38	P	5258	2048.3	15	11	4	0.732	0.294	0.438	2	N, C/N, C

^#1^ *P* papillary carcinoma, *F* follicular carcinoma.

^#2^ See Ref.^41^.

^#3^ Less than 0 in the calculation is assumed to be 0.

^#4^ *N* neck, *C* chest, *A* abdomen, *P* pelvis, *H* head.

The 30 patients with thyroid-related diseases (non-thyroid cancer) were diagnosed mainly based on pathological examination of the surgical specimens, including 14 patients with adenomatous goiter (AG), 7 with follicular adenoma (FA), and 3 with Basedow's disease. The remaining patients were diagnosed with benign diseases due to the lack of a definite pathological diagnosis (Table 2). Twenty of the 30 (67%) patients had undergone a CT examination prior to PB collection (Fig. 2A), including 2 patients who had undergone CT more than once. The body regions scanned were similar to those for the patients with thyroid cancer and were most commonly the neck and chest. The scan range was from the neck to abdomen in two patients (Table 2).

**Table 2:** Patients with thyroid related disease (non-thyroid cancer).

Case no.	Pathology^#1^ or disease	Analyzed cells number	Cell equivalent (CE)	Observed translocation (Tr)	Two-way (reciprocal) translocation	One-way (non-reciprocal) translocation	Tr frequency [/100CE]	Age correction factor^#2^	Age-adjusted Tr frequency [/100CE]^#3^	Number of CT examination before analysis	CT examination sites^#4^
1	Parathyroid adenoma	5301	2065.1	33	21	12	1.598	0.252	1.325	2	N/C, A, P
2	FA	5410	2107.5	11	9	2	0.522	0.200	0.322	1	N, C
3	FA	5327	2075.2	15	11	4	0.723	0.252	0.471	0	
4	AG	5238	2040.5	23	13	10	1.127	0.339	0.788	0	
5	Basedow's disease	5310	2068.6	5	1	4	0.242	0.309	0.000	0	
6	AG	5760	2243.9	14	8	6	0.624	0.239	0.385	1	N, C
7	AG	5393	2100.9	13	7	6	0.619	0.239	0.380	0	
8	FA	5348	2083.4	19	13	6	0.912	0.239	0.673	0	
9	AG	5262	2049.9	11	9	2	0.537	0.212	0.324	1	N, C
10	AG	5252	2046.0	12	7	5	0.587	0.339	0.247	1	N, C
11	AG	5379	2095.4	20	16	4	0.954	0.370	0.584	1	N, C
12	FA	5284	2058.4	23	16	7	1.117	0.280	0.823	1	N, C
13	Follicular lesion	5446	2121.5	24	17	7	1.131	0.294	0.822	1	N, C
14	AG	5273	2054.1	3	1	2	0.146	0.252	0.000	1	N, C, A
15	AG	5401	2104.0	23	15	8	1.093	0.280	0.813	1	N, C
16	FA	5264	2050.6	6	6	0	0.293	0.252	0.040	0	
17	Undetermined significance	5276	2055.3	21	17	4	1.022	0.294	0.714	0	
18	AG	5390	2099.7	18	17	1	0.857	0.386	0.471	1	N, C
19	FA	5336	2078.7	5	5	0	0.241	0.309	0.000	0	
20	Basedow's disease	5207	2028.4	16	11	5	0.789	0.225	0.563	1	N, C
21	FA with chronic inflammation	5258	2048.3	13	9	4	0.635	0.370	0.264	2	N, C/N, C, A
22	AG	5263	2050.3	5	2	3	0.244	0.239	0.005	1	N, C
23	AG	5233	2038.6	23	18	5	1.128	0.370	0.758	2	C/N, C
24	Follicular tumor	5248	2044.4	7	3	4	0.342	0.212	0.130	0	
25	Benign by cytology	5244	2042.9	12	6	6	0.587	0.339	0.248	0	
26	AG	5267	2051.8	17	12	5	0.829	0.339	0.490	1	N, C
27	Undetermined significance	5273	2054.1	11	7	4	0.536	0.200	0.329	1	N, C
28	Basedow's disease	5428	2114.5	13	11	2	0.615	0.239	0.376	1	N, C
29	AG	5311	2069.0	19	12	7	0.918	0.370	0.548	1	N, C
30	AG	5205	2027.7	14	10	4	0.690	0.370	0.320	1	N, C

^#1^*FA* follicular adenoma, *AG* adenomatous goiter.

^#2^See Ref.^41^.

^#3^Less than 0 in the calculation is assumed to be 0.

^#4^*N* neck, *C* chest, *A* abdomen, *P* pelvis.

Seven of the 31 (23%) individuals in the control group had undergone a CT examination prior to PB collection (Fig. 2A), of whom one had undergone CT more than once. The body regions scanned were the abdomen in two individuals and the head in five individuals; one individual underwent one abdominal CT examination because of a surgery for appendicitis in childhood, and another one individual underwent three abdominal CT examinations because of suspected ischemic enteritis due to bloody stools in childhood. The other five individuals who underwent head CT examination were for examination at the time of head contusion in childhood (Table 3).

**Table 3:** Control subjects.

Case no.	Analyzed cells number	Cell equivalent (CE)	Observed translocation (Tr)	Two-way (reciprocal) translocation	One-way (non-reciprocal) translocation	Tr frequency [/100CE]	Age correction factor^#1^	Age-adjusted Tr frequency [/100CE]	Number of CT examination before analysis	CT examination sites^#2^
1	5317	2071.3	20	8	12	0.966	0.280	0.686	1	A
3	5591	2178.0	11	2	9	0.505	0.280	0.225	0	
5	5337	2079.1	17	10	7	0.818	0.280	0.538	0	
7	5272	2053.8	15	6	9	0.730	0.280	0.450	1	H
9	5342	2081.0	16	9	7	0.769	0.294	0.475	0	
11	5301	2092.8	8	4	4	0.382	0.294	0.088	0	
13	5244	2070.3	18	14	4	0.869	0.294	0.575	0	
17	5264	2078.2	14	12	2	0.674	0.280	0.394	0	
19	5267	2079.4	11	7	4	0.529	0.294	0.235	0	
21	5250	2045.2	11	5	6	0.538	0.280	0.258	0	
23	5572	2170.6	8	3	5	0.369	0.280	0.089	0	
27	5299	2092.0	17	10	7	0.813	0.294	0.518	0	
29	5568	2198.2	17	8	9	0.773	0.294	0.479	0	
31	5263	2077.8	20	14	6	0.963	0.309	0.654	0	
35	5282	2057.7	17	9	8	0.826	0.309	0.517	0	
37	5344	2081.8	11	5	6	0.528	0.294	0.234	0	
39	5406	2106.0	11	5	6	0.522	0.280	0.242	0	
41	5288	2087.6	22	14	8	1.054	0.294	0.759	1	H
43	5297	2091.2	15	14	1	0.717	0.309	0.408	0	
45	5338	2107.4	16	10	6	0.759	0.309	0.450	0	
55	5304	2094.0	15	10	5	0.716	0.294	0.422	1	H
57	5269	2080.1	18	15	3	0.865	0.294	0.571	1	H
59	5283	2085.7	15	11	4	0.719	0.294	0.425	0	
61	5254	2074.2	32	19	13	1.543	0.294	1.248	0	
69	5263	2077.8	16	11	5	0.770	0.266	0.504	0	
71	5390	2127.9	14	10	4	0.658	0.280	0.378	0	
73	5347	2110.9	10	9	1	0.474	0.280	0.194	0	
75	5453	2152.8	7	5	2	0.325	0.280	0.045	0	
85	5355	2114.1	11	78	14	0.520	0.280	0.240	3	A
101	5317	2071.3	13	3	10	0.628	0.280	0.348	0	
117	5253	2073.8	8	6	2	0.386	0.280	0.106	1	H

^#1^See Ref.^41^.

^#2^*A* abdomen, *H* head.

### Frequency of Tr formations

**Figure 1:**
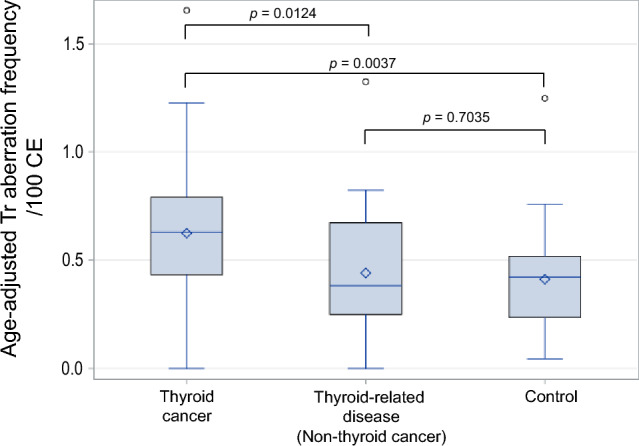
Comparison of age-adjusted translocated chromosome (Tr) frequency (per 100 cells) among three groups: thyroid cancer, thyroid-related disease (non-thyroid cancer), and controls. Significant difference was found between the thyroid cancer and thyroid-related disease groups (*p* = 0.0124) and between the thyroid cancer and control groups (*p* = 0.0037), but not between the thyroid-related disease and control groups (*p* = 0.7035). The top of the box indicates the position of 75% and the bottom the 25% of the inter-quartile range, the horizontal line inside the box indicates the median value, and the diamond indicates the mean value. The circle above is an outlier, the line above the vertical line is the largest value indicating [the top of the box + inter-quartile range × 1.5], and the line below the vertical line is the smallest value indicating [the bottom of the box + inter-quartile range × 1.5].

First, we compared the number of age-adjusted Tr aberration among the thyroid cancer, thyroid-related disease (non-thyroid cancer), and control groups. Significant difference was found between the thyroid cancer and thyroid-related diseases groups (*p* = 0.0124) and between the thyroid cancer and control groups (*p* = 0.0037), but not between the thyroid-related diseases and control groups (*p* = 0.7035) (Fig. 1).

**Figure 2:**
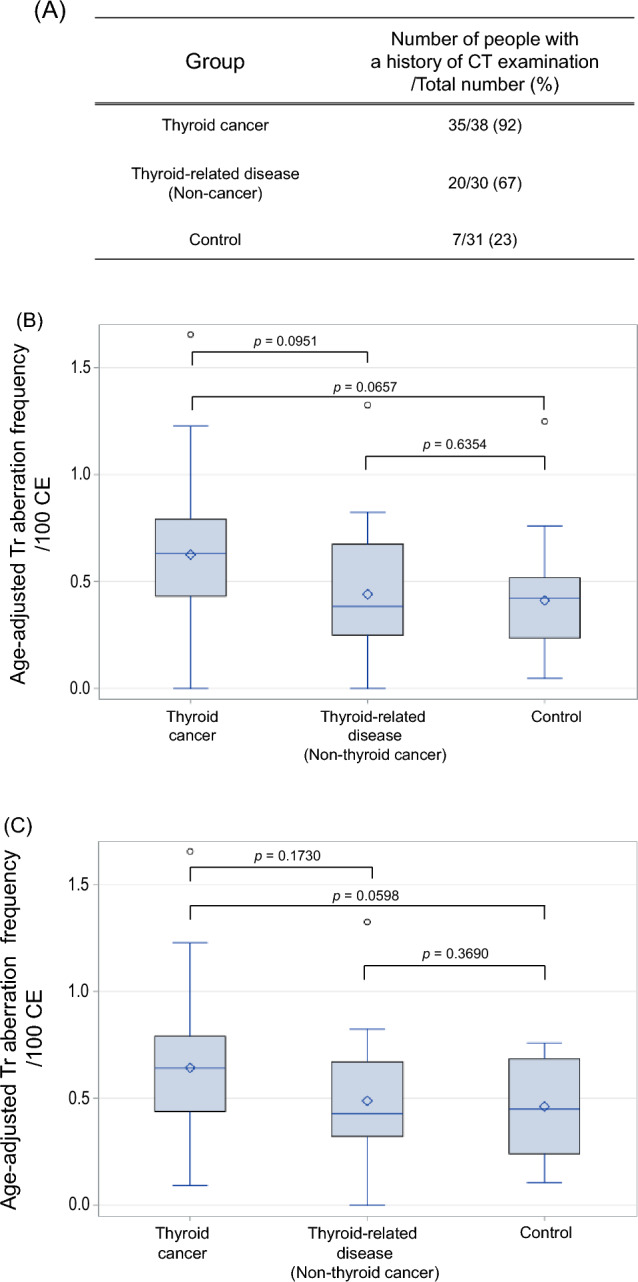
Comparison of age-adjusted Tr frequency (per 100 cells) among the three groups after adjustment for sex and a history of CT examination. (**A**) Number of individuals in each group with a history of CT examination. (**B**) After adjusting for sex and a history of CT examination, comparison of age-adjusted Tr frequency showed no significant difference between the thyroid cancer and thyroid-related disease groups (*p* = 0.0951), between the thyroid cancer and control groups (*p* = 0.0657), and between the thyroid-related disease and control groups (*p* = 0.6354). (**C**) In those who had undergone CT examination, comparison of age-adjusted Tr frequency after adjustment for sex showed no significant difference between the thyroid cancer and thyroid-related disease groups (*p* = 0.1730), between the thyroid cancer and control groups (*p* = 0.0598), and between the thyroid-related disease and control groups (*p* = 0.3690). See note on Fig. 1 for figure description.

Second, there was no significant difference in age-adjusted Tr aberration frequency between males and females (Supplementary Fig. S1). In patients with thyroid cancer or non-thyroid cancer, CT examination of the neck and chest was performed as part of the standard pre-surgical workup, particularly in those with thyroid cancer, 92% of whom underwent CT (Fig. 2A).

In our previous study, we reported chromosome aberrations due to radiation exposure during a single CT scan^17–19^, and recommended that the effects of CT examination should be considered, especially in children. In fact, there was a significant difference in age-adjusted Tr aberration frequency between those who did and did not undergo CT examination (Supplementary Fig. S2). Therefore, although there was no significant difference in age-adjusted Tr aberration frequency between males and females, we compared the frequency (per 100 cells) among the thyroid cancer, thyroid-related disease (non-thyroid cancer), and control groups after adjusting for sex and history of CT examination. The results showed no significant difference among the three groups (Fig. 2B). Furthermore, we found no significant difference in frequency when we compared age-adjusted Tr aberration frequency among those with a history of CT examination (Fig. 2C).

In conclusion, a comparison of age-adjusted Tr aberration frequency among the three groups of thyroid cancer patients, thyroid-related disease (non-thyroid cancer) patients, and the controls revealed a slight increase in age-adjusted Tr formation in patients with thyroid cancer, but the difference was not significant after adjusting for sex and a history of CT examinations. These findings suggest that CT examinations performed prior to treatment may have affected Tr formation in patients with thyroid cancer.

We also compared Tr frequency before age-adjustment among the same three groups as above, and similar results were obtained (Supplementary Fig. S3).

## Discussion

The purpose of this study was to investigate whether radiation exposure due to the nuclear accident is the cause of the higher number of thyroid cancer cases found in the pediatric TUE program in FP compared with the estimated number from the cancer registry^6^. More than 10 years after the GEJE, the relationship between the occurrence of thyroid cancer and thyroid equivalent dose has not been fully analyzed, for the reason that estimated individual thyroid equivalent doses based on the behavioral survey for the 4 months immediately after the GEJE conducted by FP^20^ have only been analyzed for about 40% of patients with thyroid cancer^14^. Alternatively, effective doses above 250 mSv can be estimated based on the number of Tr formations using PB lymphocytes^15^. However, unlike analysis of Dic formation, which is used to estimate dose in the acute phase after radiation exposure, analysis of Tr formation is susceptible to long-term effects such as smoking, drugs, and natural radiation. Our previous studies revealed an increase in Dic formations after a single CT scan^17^, but it was impossible to find a significant increase in Tr formations^19^, which theoretically are produced in about an equal ratio to Dic formations^21,22^, and we did not find an accumulative increase in Tr formations after three consecutive CT scans^18^. It is thought that the reason for these findings is the influence of the above confounding factors, especially in subjects who are middle-aged or older. Conversely, the present subjects were young (mean age, approximately 20 years), and we therefore considered the effect of confounding factors to be small. If the Tr formations in the present study were caused mainly by radiation exposure, the majority might have been exposed to about 100 mSv, regardless of their history of CT examination based on the calibration curves of our 23-year-old healthy subjects^16^, which is too high when compared to the external exposure dose estimated from the behavioral survey^23,24^. As we have learned from our dose–response curves^16^, there are age-related differences in the Tr formation even in healthy subjects, which accordingly suggests that a dose–response curve for at least every 10 years might be necessary. Therefore, it is difficult to determine radiation exposure below 100 mSv based on the number of Tr formations.

A significant feature of the present study is that rather than performing a comparison between two groups (thyroid cancer and healthy control groups), Tr aberration frequency was compared among three groups, and included patients with benign thyroid tumors and other thyroid-related diseases other than thyroid cancer. Initially, Tr aberration frequency was significantly higher in patients with thyroid cancer (Fig. 1), which we suspected was due to genetic instability, as has been proposed for many cancer diseases^25^. In fact, the thyroid cancer found in patients in Fukushima was reported to have a different genetic abnormality to those in Chernobyl^26,27^. However, there was a significant difference in Tr aberration frequency between patients with and without a history of CT examination (Supplementary Fig. S2), and the difference was no longer statistically significant after adjusting for sex and a history of CT examination (Fig. 2B). In addition, comparison of Tr aberration frequency among only those subjects with a history of CT examination showed no significant difference (Fig. 2C).

Almost all of patients with thyroid cancer or with a benign thyroid tumors had underwent CT examination prior to surgical treatment of the thyroid gland, which was mainly CT of the neck and chest. As in our previous studies^17,19^, in which subjects were analyzed using the WAZA-ARI system, we estimated the effective dose^28–30^ as 7.1 ± 1.3 (mean ± standard deviation) mSv (n = 8) for CT of the neck, 22.8 ± 9.4 mSv (n = 4) for CT of the chest, and 54.5 ± 7.9 mSv (n = 3) for CT from the chest to pelvis. Furthermore, the effective dose for the head using the same CT equipment as above was approximately 1.8 mSv for a standard Japanese body type of adult. Although WAZA-ARI could have been used to estimate past exposure doses based on the specific detailed of each CT scanning site, in the present study we did not consider differences in the number of CT examinations or CT scanning sites for the following reasons: (1) CT equipment differed between facilities; (2) some thyroid cancer and non-thyroid cancer patients, including some of the control subjects, had multiple CT examinations; (3) effective dose of head CT, which was more frequently performed in the control subjects, is lower than those for other CT scanning sites by WAZA-ARI; and (4) estimation of radiation exposure dose below 100 mSv based on the number of Tr formation is difficult, as mentioned above.

If the initial higher Tr aberration frequency in the thyroid cancer group had been caused by radiation exposure from the nuclear power plant accident, then the frequency should have remained significantly higher in that group even after adjusting for a history of CT examination. However, the significant difference among the three groups disappeared after adjusting for a history of CT examination. Furthermore, the subjects in this study were younger than 30 years of age, and the effect of confounding factors is considered small. Therefore, we consider the CT examinations to have the greatest impact on the occurrence of chromosomal translocations. Accordingly, we consider that radiation exposure by CT examination is the most likely cause of the increased Tr aberration frequency.

Unfortunately, external exposure doses based on behavioral surveys have been obtained in only about 28% of residents of FP^23,24^, and thus there were insufficient such results in the present subjects for inclusion in this study. However, in more than 97% of the evacuees who do have behavioral survey records, the external exposure dose has been reported as 3 mSv or less^23^, and an analysis of the representativeness of individual external doses has indicated that if they lived in the same area where the residents with known exposure doses lived at the time of the GEJE, their exposure dose levels were the same as those of residents with known exposure doses^31^. Therefore, as already reported, no relationship has been found between the occurrence of pediatric thyroid cancer in FP and external exposure doses, absorbed doses in the thyroid gland, or place of residence^5,7–11^. The results of this study support the claim that the occurrence of pediatric thyroid cancer in FP was not caused by radiation exposure due to the nuclear accident.

In contrast, the present results suggest that CT examination in young people aged ≤ 20 years can induce an increase in lymphocytes with Tr formation in PB. Previous studies have reported that CT examinations in children increased the risk of cancer development (especially brain tumors)^32–34^, suggesting the possibility that DNA or chromosome damage due to radiation exposure by CT examination might be responsible for inducing chromosomal abnormalities or genetic mutations. However, Pearce MS et al.’s report did not state why those children needed to undergo CT examinations^32^. On the other hand, when children with cancer-predisposing factors (PFs) such as congenital genetic abnormalities and immunodeficiency were excluded, there was no relationship between CT examination and risk of developing cancer^34^. This might suggest, conversely, that children with PFs would be at risk of cancer development by CT examinations. A recent study has reported that radiation dose from head and neck CT examinations before the age of 22 is associated with the subsequent development of brain tumors^35^. The study had a 5-year observation period after the CT examination to rule out latent cancer at the time of the CT scan, and it is of interest that the excess relative risk (ERR) of brain tumor was highest in 5 to > 10 years and lowest in $\ge$ 15 years since the exposure. Therefore, it is important to minimize medical exposures from such as CT examination, especially in young people.

In conclusion, the analysis of Tr aberration frequency using PB lymphocytes, an indicator of radiation exposure, supports the claim that there was no evidence of additional exposure without CT examinations in pediatric thyroid cancer patients compared with the control. Furthermore, it should be noted that medical exposure from CT scans in children and adolescents can affect chromosomes or genes.

## Patients and methods

### Ethics statement

The use of samples and the medical records in this study was approved by the Ethics Committee of the Fukushima Medical University School of Medicine (approval No. 2654). Written informed consent was obtained from all participants for analysis of their PB samples, and the protocols were carried out in accordance with approved guidelines of the Council for International Organizations of Medical Science^36^.

### Subjects

Residents under the age of 18 in FP at the time of the GEJE undergo the TUE (primary examination). If nodules are found, a more detailed TUE (confirmatory examination) is performed, and if necessary, fine needle aspiration cytology (FNAC) according to the guidelines for thyroid diseases^7,37,38^. If diagnosed with cancer, the patient undergoes surgical treatment of the tumor according to the guidelines for thyroid diseases^5,7^. Initially, 40 patients with thyroid cancer were enrolled, but one was excluded because of a history of radiotherapy and chemotherapy for a brain tumor. Another one patient was excluded because the patient was found outside of FP and was unrelated to the TUE program in FP. As controls, we enrolled 32 healthy students over 20 years of age from Fukushima Medical University, but one patient with a history of CT examination was excluded because of a history of surgery for a cervical tumor in childhood.

Three groups were included in the analyses: the thyroid cancer group (n = 38; 15 males, 23 females; age range 12–26 years; median age, 18 years), the thyroid-related disease (non-thyroid cancer) group (n = 30; 6 males, 24 females; age range 15–28 years; median age, 21 years), and the control group (n = 31; 20 males, 11 females; age range 20–23 years; median age, 22 years). The number of CT examinations and examination sites prior to sample collection are shown for patients with thyroid cancer, patients with thyroid-related diseases (non-thyroid cancer), and individuals of the control group in Tables 1, 2, and 3, respectively. Age information is not included in those tables to protect individual information.

### Separation of lymphocytes from PBs and cell culture conditions

Mononuclear blood cells were isolated from heparinized PBs using BD Vacutainer CPT tubes (BD Biosciences, San Jose, CA, USA) according to the manufacturer’s instructions. Cells were suspended in RPMI 1640 medium (Nacalai Tesque, Kyoto, Japan) containing 20% fetal bovine serum (Equitech Bio, Keilor East, Australia), 2% phytohaemagglutinin-HA15 (Remel, Lenexa, KS, USA), and 60 μg/mL of kanamycin solution (Life Technologies, Carlsbad, CA, USA) in a 15-mL Falcon tube. Lymphocytes were cultured in a 5% humidified CO_2_ incubator at 37 °C for 48 h. First-division metaphase cells were obtained by treating the culture with colcemid (final concentration, 0.05 μg/mL; Life Technologies) for 48 h. For samples younger than 18 years, colcemid was added for the last 24 h to prevent overcontraction.

#### Cell harvesting

After 48 h of culture, cells were harvested, treated with 0.075 M KCL, and fixed with methanol/acetic acid (3:1) according to the standard cytogenetic procedure^15,39^. Finally, the cell pellets were suspended in 1–2 mL of fixative, depending on the size of the pellets. One drop (around 20 μL) of the suspension was dispensed onto a slide and spread on a water bath.

#### Chromosome painting

Each slide was first dried at 65 °C for at least 1 h for hardening. Next, 6–7 μL of a Customized XCP-Mix probe (Mix-#1R-#2G-#4RG; MetaSystems, Altlussheim, Germany) solution was applied per 22 × 22-mm area, and the slide was covered with a glass coverslip and sealed with paper bond. Subsequent operations were carried out according to the manufacturer’s instructions. Nuclear DNA was denatured by incubating the slides on a hot plate at 75 °C for 2 min followed by incubation overnight at 37 °C in a humidified chamber to allow for hybridization. The glass coverslips were removed and the slides were washed in 0.4 × SSC at 72 °C for 2 min. After draining, the slides were then washed in 2 × SSC/0.05% Tween-20 at room temperature (RT) for 30 s. Subsequently, the slides were briefly rinsed in distilled water to avoid crystal formation and then air dried at RT. Finally, nuclei were counterstained with Vectashield Mounting Medium containing DAPI (Vector, Burlingame, USA), and the slides were covered with a glass coverslip and sealed with nail polish.

#### Image capturing and scoring of chromosomal aberrations (chromosome 1, 2, and 4 painting)

Soon after completion of the chromosome preparations, FISH images were captured in AutoCapt mode using two sets of AXIO Imager Z2 microscopes (Carl Zeiss AG, Oberkochen, Germany) equipped with CCD cameras and Metafer 4 software (MetaSystems GmbH, Altlussheim, Germany). Metaphase cells were selected for scoring in manual mode. Chromosome analysis was performed according to the International Atomic Energy Agency (IAEA) manual (IAEA 2001)^39^ by a trained, experienced observer blinded to information of the subjects’ backgrounds.

In our previous study, we analyzed Dic formation using Giemsa staining and centromere-FISH, and analyzed 2000 metaphases per patient^17–19^. Accordingly, to match the number of cells analyzed between the Dic and Tr analyses, we analyzed approximately 5000 cells, which was equivalent to whole-genome analysis of almost 2000 cells (Tables 1, 2, and 3, respectively.).

Only metaphase figures with about 44–46 chromosomes were selected for chromosomal analysis. Some clonal chromosome aberrations were suspected, but we did not correct the number of Tr accordingly. Thus, cells with three chromosome (1, 2, and 4) pairs colored in three different paintings were selected for analysis. Metaphase cells exhibiting tetraploidy were omitted from the analysis. When no translocated chromosome partner was found, it was counted as one-way (non-reciprocal); when the partner was found, it was counted as two-way (reciprocal). And in both cases, it was counted as one translocated chromosome. In the case of complex chromosomal abnormalities, the translocation number was determined based on the number of color junctions (NCJ)^40^. For example, an NCJ of 1 or 2 indicates one translocation, an NCJ of 3 or 4 indicates two translocations, an NCJ of 5 or 6 indicates three translocations, and so on.

For scoring, the formula used to calculate the frequency of translocations across the whole genome (F_G_) was based on the following formula, using three colors (chromosome 1, red; chromosome 2, green; chromosome 4, yellow) for the detected translocations:

$$ {\text{F}}_{{\text{G}}} = {\text{ F}}_{{{\text{P}}({1} + {2} + {4})}} /{ 2}.0{5 }\left[ {{\text{f}}_{{1}} \left( {{1}{-}{\text{f}}_{{1}} } \right) + {\text{f}}_{{2}} \left( {{1}{-}{\text{f}}_{{2}} } \right) + {\text{f}}_{{4}} \left( {{1}{-}{\text{f}}_{{4}} } \right) \, {-} \, \left( {{\text{f}}_{{1}} {\text{f}}_{{2}} + {\text{f}}_{{1}} {\text{f}}_{{4}} + {\text{f}}_{{2}} {\text{f}}_{{4}} } \right)} \right] $$

*F*_*G*_: the full genome aberration frequency, *F*_*p*_: the translocation frequency detected by FISH, *f*_*p*_: the fraction of genome hybridized, taking into account the sex of the subject (female:* f*_*p*_ = 0.2234, male: *f*_*p*_ = 0.2271).

The proportion of the genome occupied by chromosomes 1, 2, and 4 is about 23%. Therefore, F_G_ is determined by the following formula:

$$ \begin{gathered} {\text{F}}_{{\text{G}}} = {\text{ F}}_{{\text{P}}} \times {2}.{567 }\left( {{\text{female}}} \right) \hfill \\ {\text{F}}_{{\text{G}}} = {\text{ F}}_{{\text{P}}} \times {2}.{533 }\left( {{\text{male}}} \right) \hfill \\ \end{gathered} $$

To unify the cell numbers of the analysis, we determined F_G_ as per the 2000-cell equivalents, which were obtained according to the above formulas for females and males, respectively. In addition, age-adjusted Tr frequency was determined based on the method of Sigurdson et al.^41^.

### Statistical analysis

First, differences in age-adjusted Tr aberration frequency between those with and without a history of CT examination and between men and women were analyzed by Student's t-test. Differences in age-adjusted Tr aberration frequency among patients with thyroid cancer, thyroid-related disease (non-thyroid cancer), and controls were then tested using ANOVA. If the test by ANOVA was significant, differences between groups were compared by Tukey's test. Because a history of CT examination affected the age-adjusted Tr frequency, we made the same comparisons only among those with a history of CT scan. In addition, differences in age-adjusted Tr aberration frequency among patients with thyroid cancer, thyroid-related disease (non-thyroid cancer), and controls were compared by ANCOVA after adjusting for sex and history of CT examination. SAS version 9.4 (SAS Institute, Cary, NC, USA) was employed for all statistical analyses, with two-tailed probability values for the statistical tests. *P* values less than 0.05 were considered statistically significant.

### Supplementary Information

Supplementary Information 1.

Supplementary Information 2.

Supplementary Information 3.

Supplementary Information 4.

## Data Availability

The datasets generated and/or analyzed during the current study are not publicly available due [Even in Fukushima Prefecture, the number of thyroid cancer patients is limited, and there is a risk that individuals could be identified, which could lead to harmful rumors, so the information cannot be disclosed. In fact, some patients have legal battles with the Fukushima nuclear power plant company] but are available from the corresponding author on reasonable request.
