# Supplementary figures and images for: No increase in translocated chromosomal aberrations, an indicator of ionizing radiation exposure, in childhood thyroid cancer in Fukushima Prefecture
Source: Sci Rep. 2023 Aug 31;13:14254. doi: 10.1038/s41598-023-41501-x (PMC10471584; doi:10.1038/s41598-023-41501-x)

## Slide 1
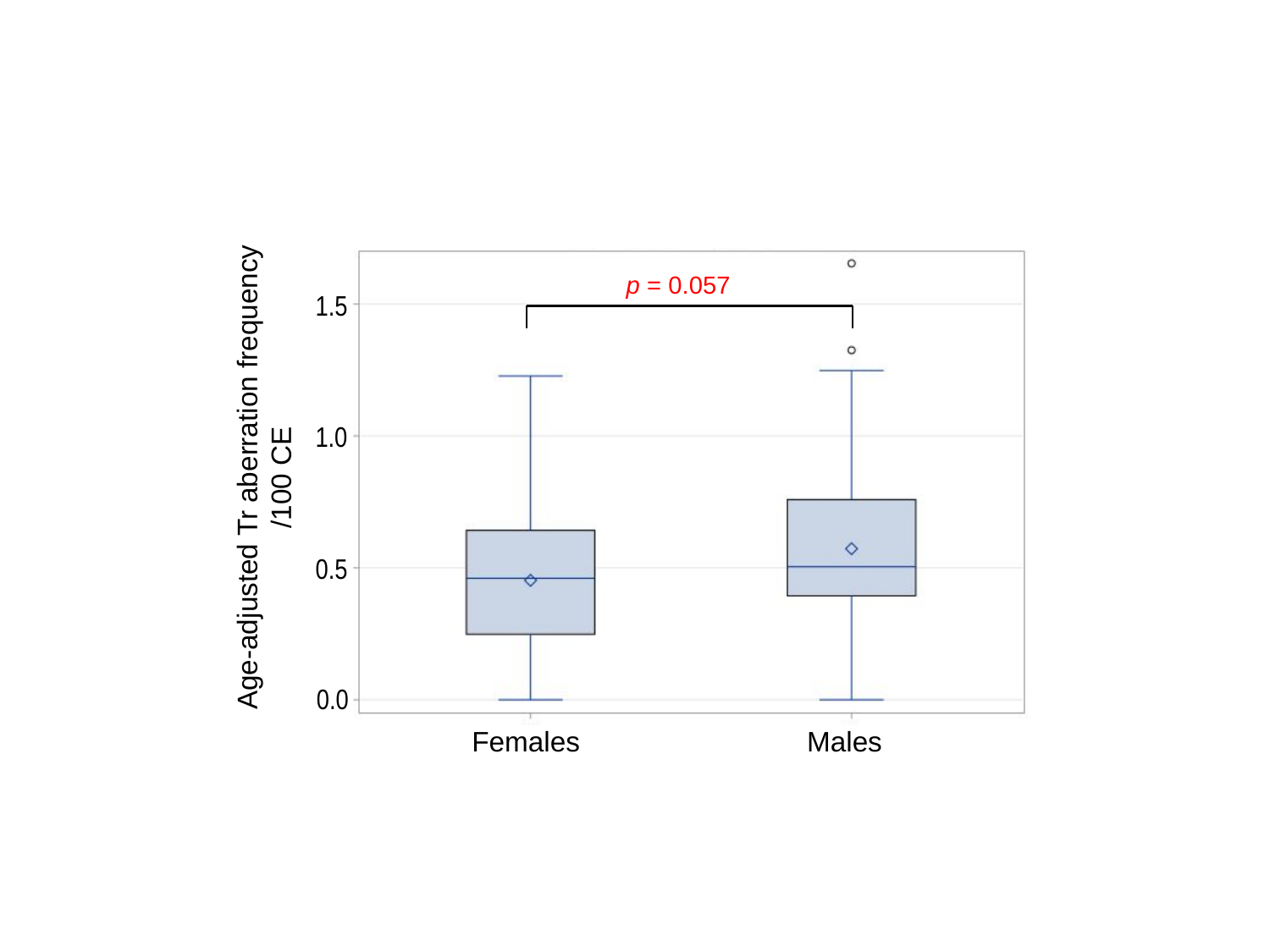

p = 0.057
1.5
1.0
Age-adjusted Tr aberration frequency
/100 CE
0.5
0.0
Males
Females

Supplement: Supplementary file 1 — Supplementary Information 1. [file 41598_2023_41501_MOESM1_ESM.pptx]

## Slide 1
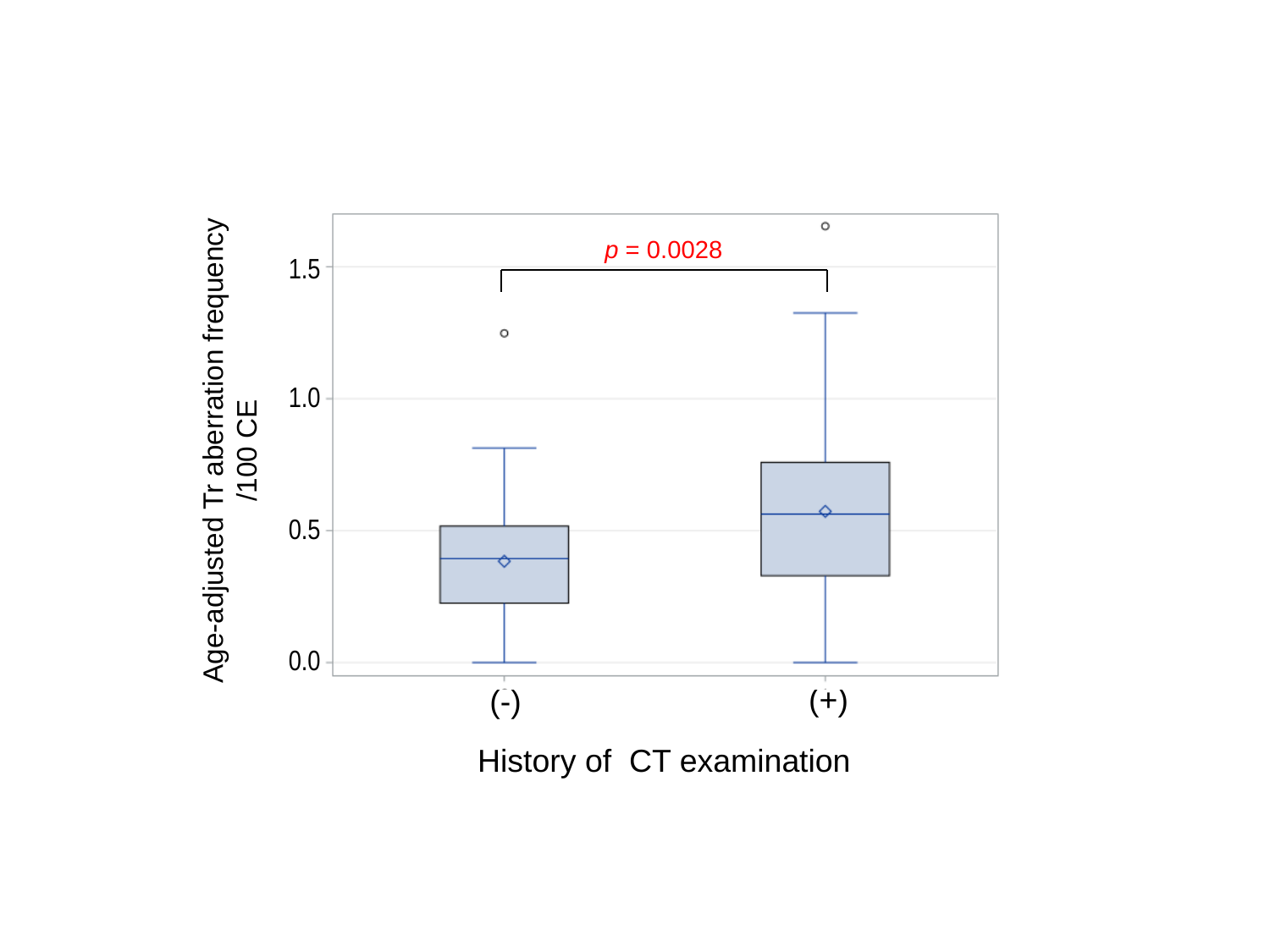

1.5
1.0
Age-adjusted Tr aberration frequency
/100 CE
0.5
0.0
p = 0.0028
(+)
(-)
History of CT examination

Supplement: Supplementary file 2 — Supplementary Information 2. [file 41598_2023_41501_MOESM2_ESM.pptx]
