# Supplementary material for: No increase in translocated chromosomal aberrations, an indicator of ionizing radiation exposure, in childhood thyroid cancer in Fukushima Prefecture
Source: Sci Rep. 2023 Aug 31;13:14254. doi: 10.1038/s41598-023-41501-x (PMC10471584; doi:10.1038/s41598-023-41501-x)
Supplement: Supplementary file 3 — Supplementary Information 3. [file 41598_2023_41501_MOESM3_ESM.pptx]

## Slide 1
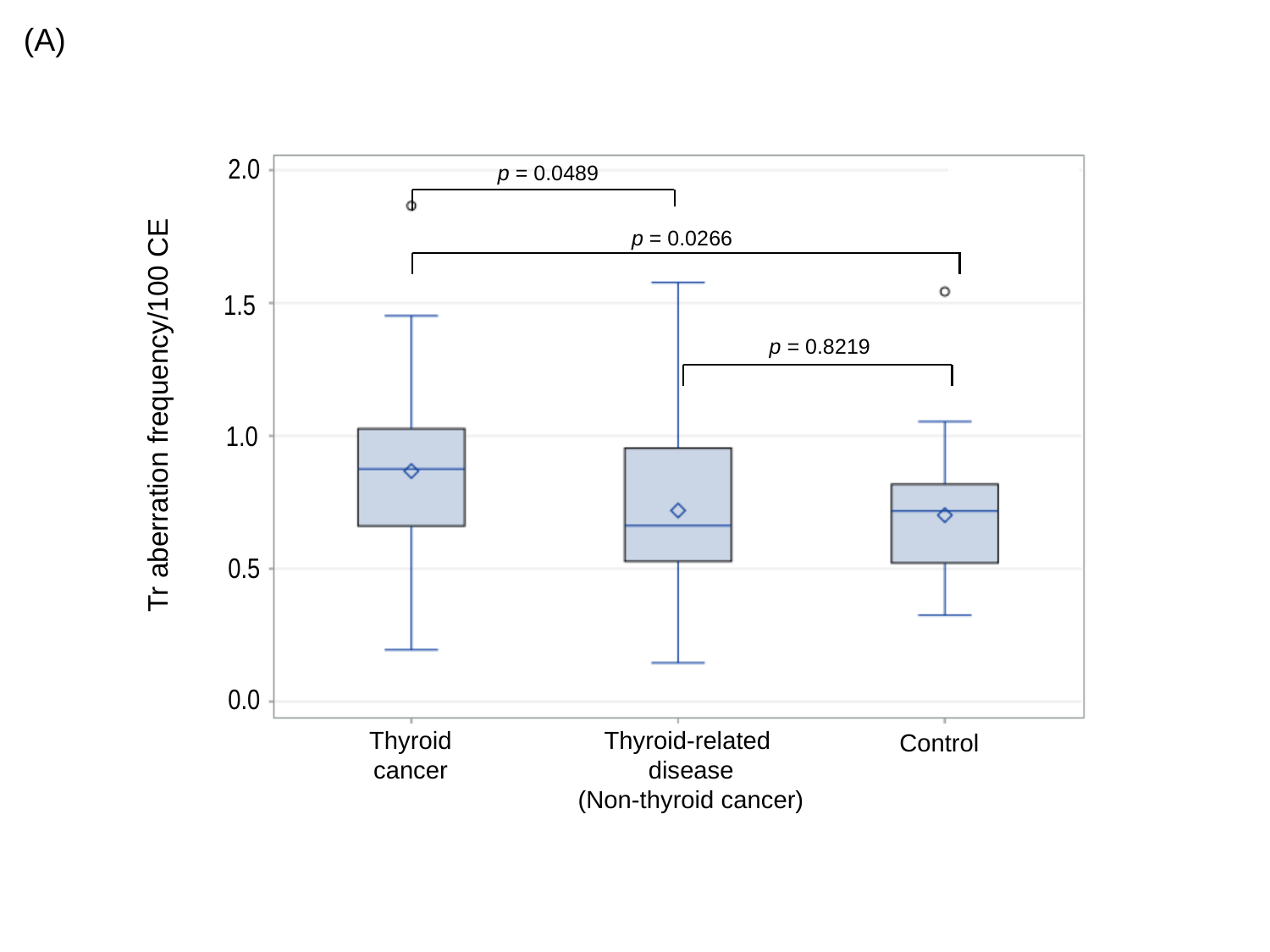

(A)
2.0
p = 0.0489
p = 0.0266
1.5
p = 0.8219
1.0
0.5
0.0
Thyroid
cancer
Thyroid-related
disease
(Non-thyroid cancer)
Control
Tr aberration frequency/100 CE

## Slide 2
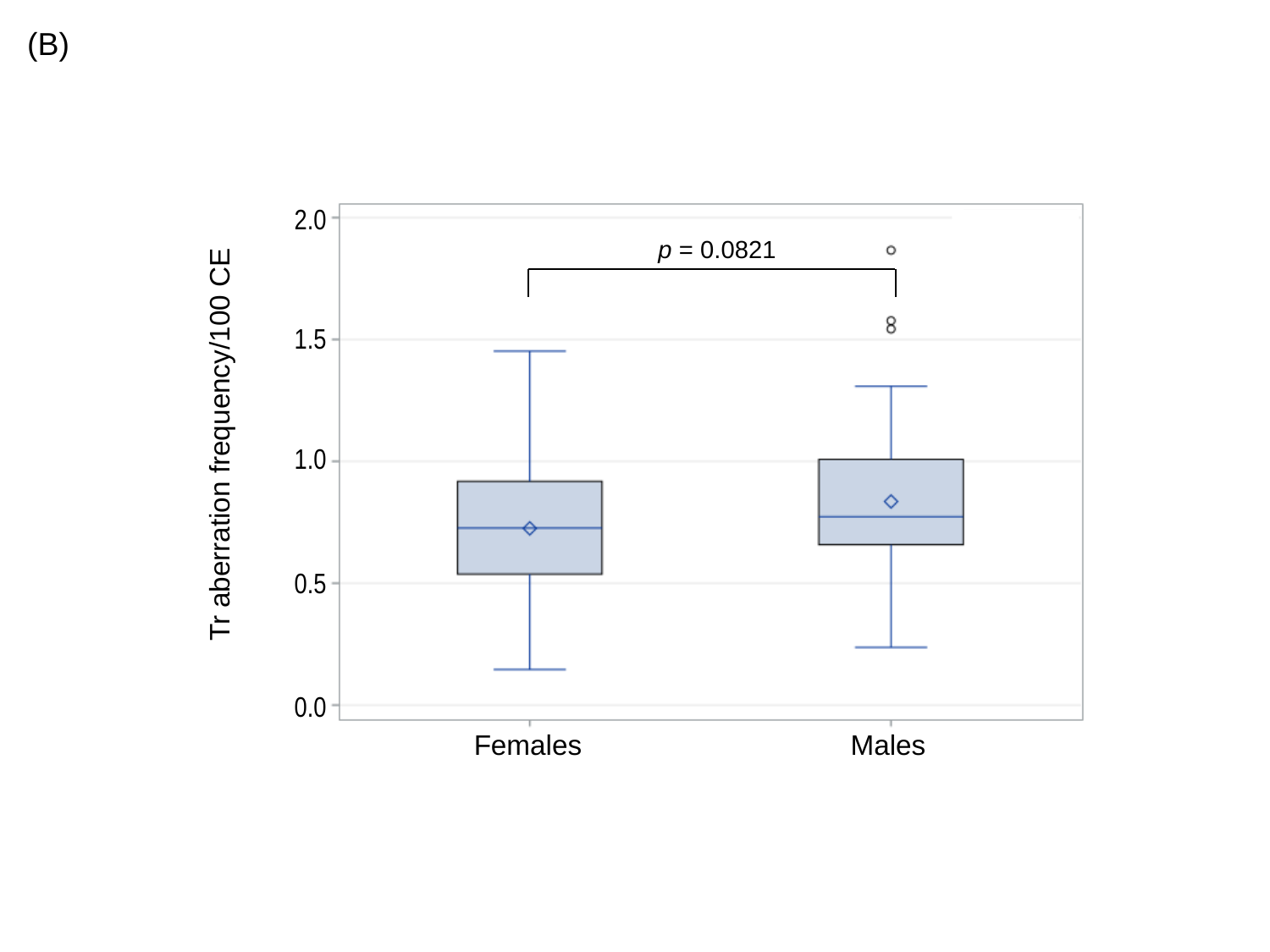

(B)
2.0
p = 0.0821
1.5
Tr aberration frequency/100 CE
1.0
0.5
0.0
Females
Males

## Slide 3
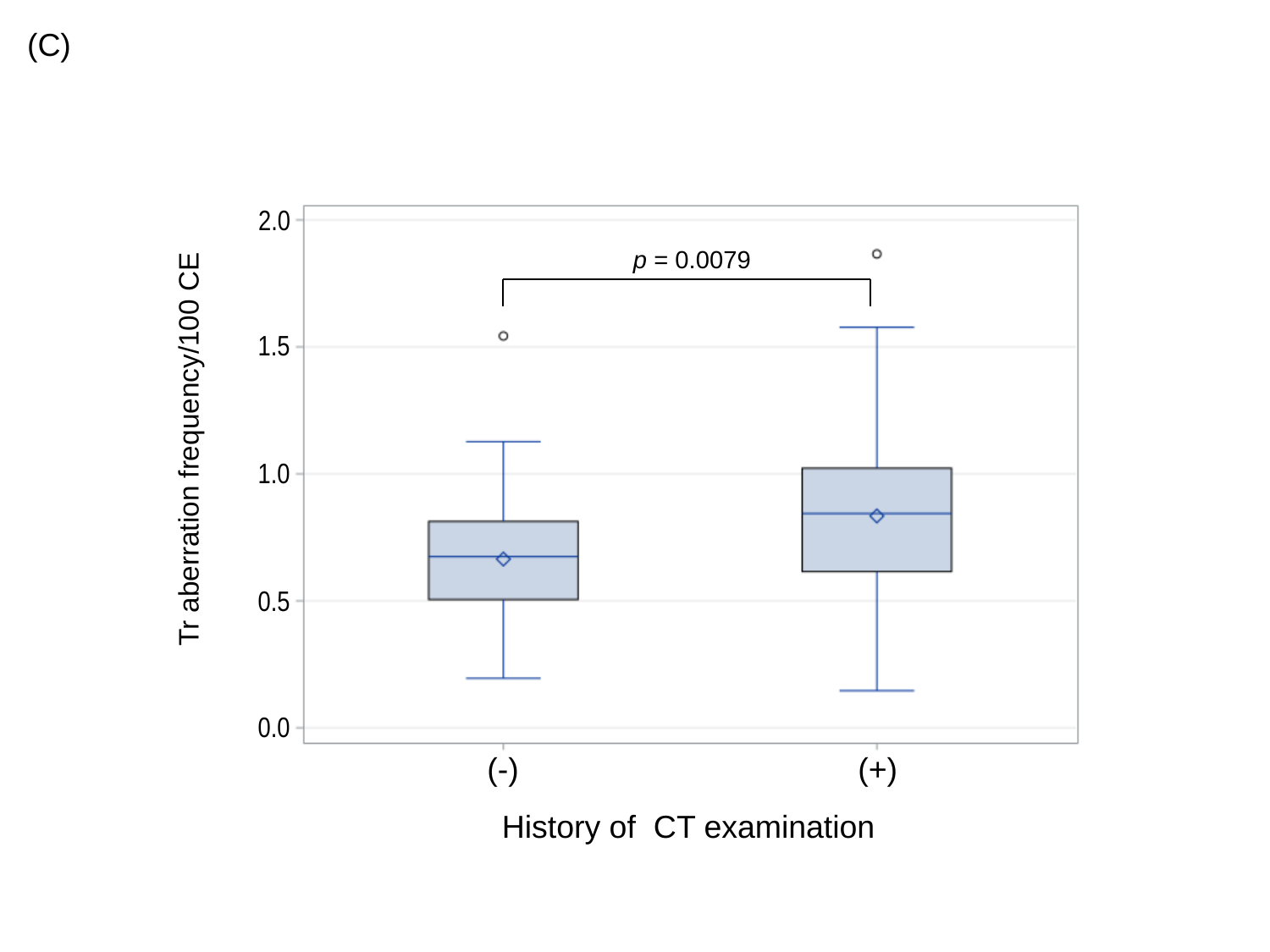

(C)
2.0
p = 0.0079
1.5
Tr aberration frequency/100 CE
1.0
0.5
0.0
(-)
(+)
History of CT examination

## Slide 4
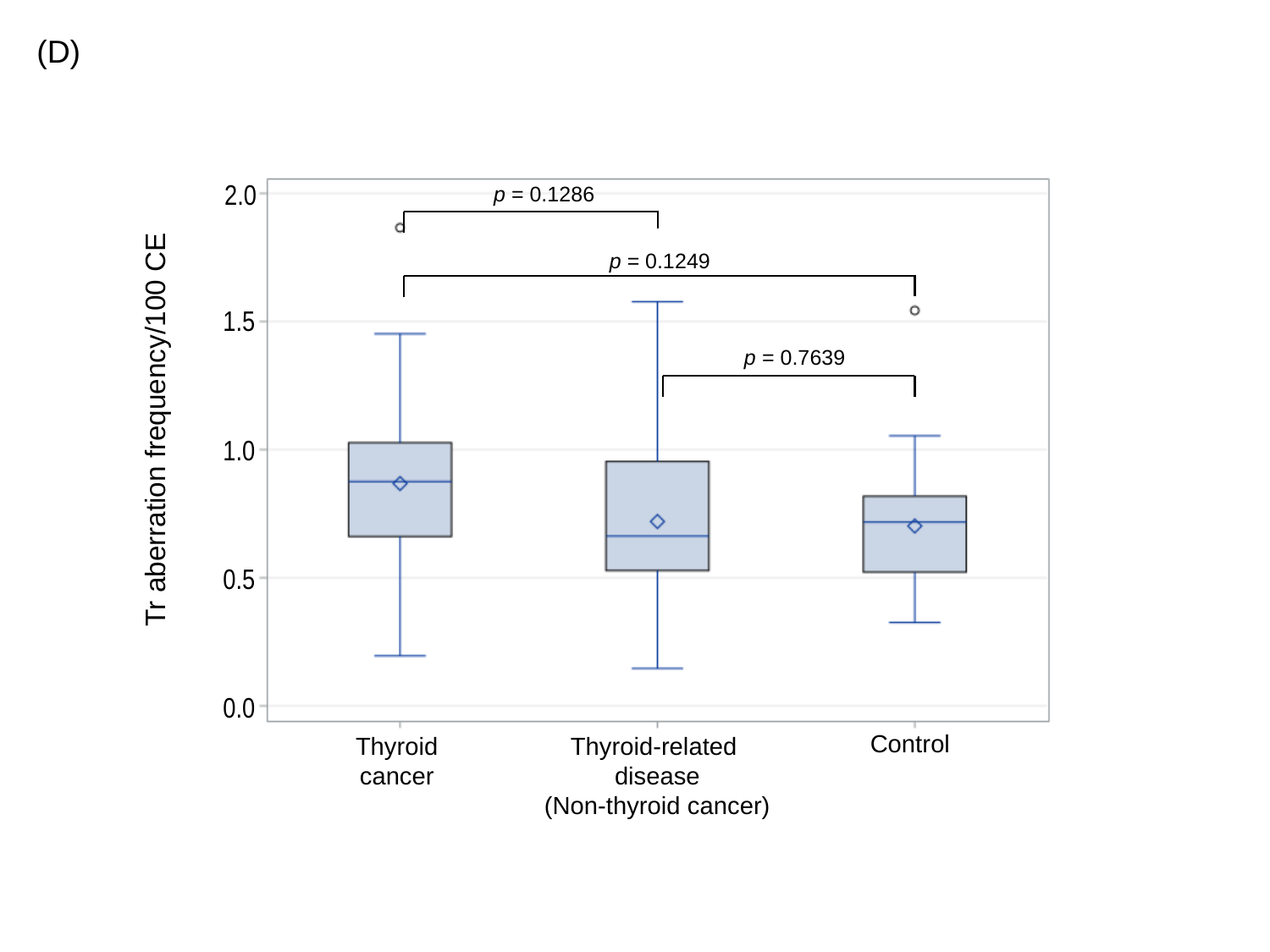

(D)
2.0
p = 0.1286
p = 0.1249
1.5
p = 0.7639
Tr aberration frequency/100 CE
1.0
0.5
0.0
Control
Thyroid-related
disease
(Non-thyroid cancer)
Thyroid
cancer

## Slide 5
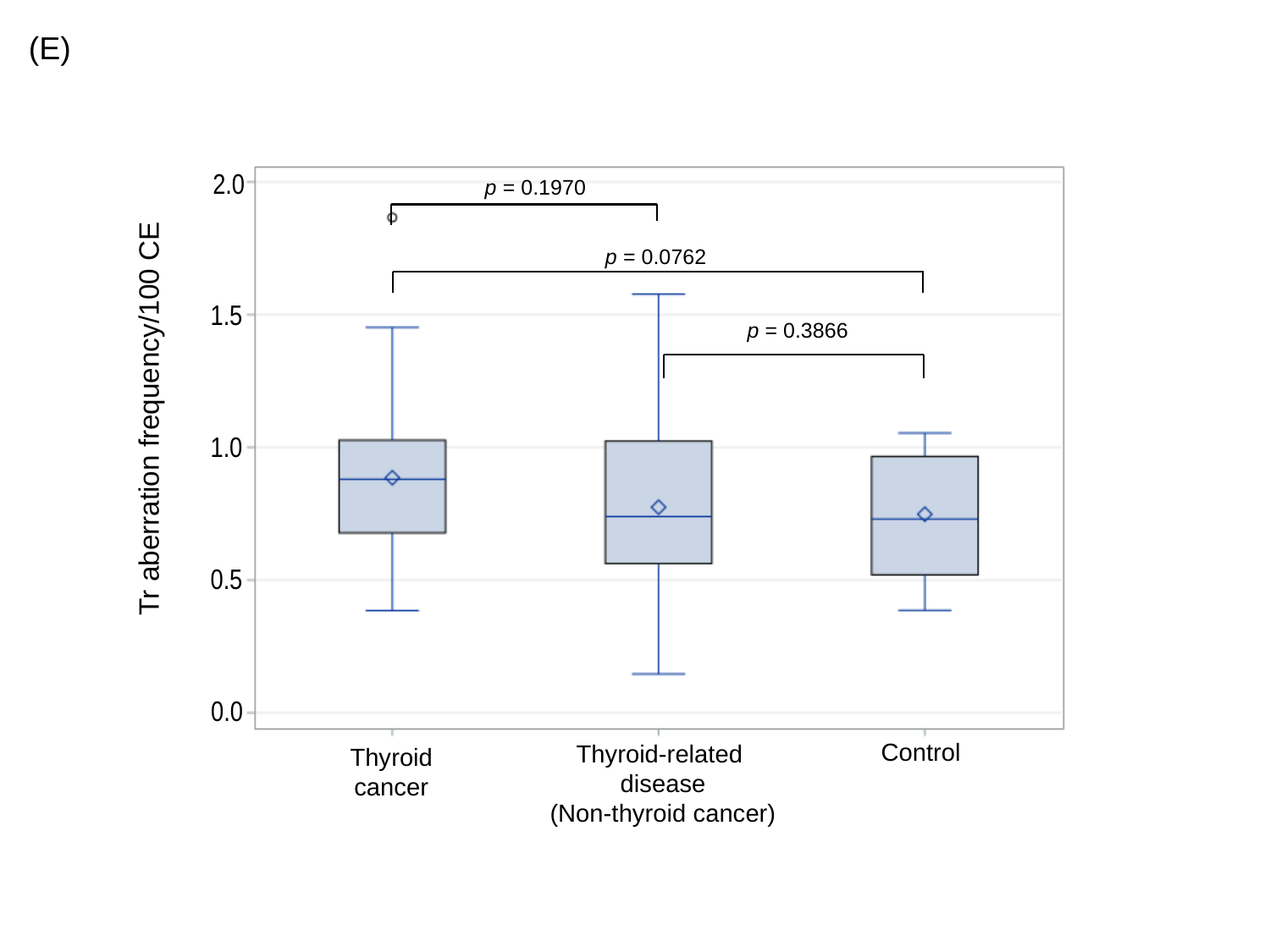

(E)
2.0
p = 0.1970
p = 0.0762
1.5
p = 0.3866
Tr aberration frequency/100 CE
1.0
0.5
0.0
Control
Thyroid-related
disease
(Non-thyroid cancer)
Thyroid
cancer
