# Supplementary material for: No increase in translocated chromosomal aberrations, an indicator of ionizing radiation exposure, in childhood thyroid cancer in Fukushima Prefecture
Source: Sci Rep. 2023 Aug 31;13:14254. doi: 10.1038/s41598-023-41501-x (PMC10471584; doi:10.1038/s41598-023-41501-x)
Supplement: Supplementary file 4 — Supplementary Information 4. [file 41598_2023_41501_MOESM4_ESM.docx]

**Supplementary information**

**Supplementary figure 1**. **Comparison of age-adjusted translocated chromosome (Tr) frequency (per 100 cells) according to sex.**

There was no significant difference in age-adjusted Tr frequency (per 100 cells) by sex (*p* = 0.057).

The top of the box indicates the position of 75% and the bottom the 25% of the inter-quartile range, the horizontal line inside the box indicates the median value, and the diamond indicates the mean value. The circle above is an outlier, the line above the vertical line is the largest value indicating [the top of the box + inter-quartile range × 1.5], and the line below the vertical line is the smallest value indicating [the bottom of the box + inter-quartile range × 1.5].

**Supplementary figure 2**. **Comparison of age-adjusted Tr frequency (per 100 cells) according to history of CT examination.**

Age-adjusted Tr frequency (per 100 cells) was significantly higher in individuals with a history of CT examination than in those without a history of CT examination (*p* = 0.0028).

See note on Supplementary figure 1 for figure description.

**Supplementary figure 3. Analysis of Tr frequency (per 100 cells) without the method of Sigurdson et al.**

**(A)** Comparison of Tr frequency (per 100 cells) among three groups: thyroid cancer, thyroid-related disease (non-thyroid cancer), and controls. Significant difference was found between the thyroid cancer and thyroid-related disease groups (*p* = 0.0489) and between the thyroid cancer and control groups (*p* = 0.0266), but not between the thyroid-related disease and control groups (*p* = 0.8219). **(B)** Comparison of Tr frequency (per 100 cells) according to sex. There was no significant difference in Tr frequency (per 100 cells) by sex (*p* = 0.0821). **(C)** Comparison of Tr frequency (per 100 cells) according to a history of CT examination. Tr frequency (per 100 cells) was significantly higher in individuals with a history of CT examination than in those without a history of CT examination (*p* = 0.0079). **(D)** Comparison of Tr frequency (per 100 cells) among the three groups after adjustment for sex, age, and a history of CT examination. After adjusting for sex, age, and a history of CT examination, comparison of Tr frequency showed no significant difference between the thyroid cancer and thyroid-related disease groups (*p* = 0.1286), between the thyroid cancer and control groups (*p* = 0.1249), and between the thyroid-related disease and control groups (*p* = 0.7639). **(E)** In those who had undergone CT examination, comparison of Tr frequency after adjustment for sex and age showed no significant difference between the thyroid cancer and thyroid-related disease groups (*p* = 0.1970), between the thyroid cancer and control groups (*p* = 0.0762), and between the thyroid-related disease and control groups (*p* = 0.3866).

See note on Figure 1 for figure description.
